# Clinical effects of continuous veno-venous hemofiltration combined with hemoperfusion for the treatment of multiple myeloma complicated with acute kidney injury

**DOI:** 10.12669/pjms.39.2.6966

**Published:** 2023

**Authors:** Jing Huang, Fengping Qiu, Huiqi Zhang, Xiangli Shen, Xia Lin

**Affiliations:** 1 Jing Huang, Department of Hematology, The First People’s Hospital of Huzhou, Zhejiang Province, Huzhou 313000, Zhejiang Province, P.R. China; 2 Fengping Qiu, Department of Nephrology, The First People’s Hospital of Huzhou, Zhejiang Province, Huzhou 313000, Zhejiang Province, P.R. China; 3 Huiqi Zhang, Department of Hematology, The First People’s Hospital of Huzhou, Zhejiang Province, Huzhou 313000, Zhejiang Province, P.R. China; 4 Xiangli Shen, Department of Hematology, The First People’s Hospital of Huzhou, Zhejiang Province, Huzhou 313000, Zhejiang Province, P.R. China; 5 Xia Lin, Department of Hematology, The First People’s Hospital of Huzhou, Zhejiang Province, Huzhou 313000, Zhejiang Province, P.R. China

**Keywords:** Acute kidney injury, Continuous veno-venous hemofiltration, Hemoperfusion, Multiple myeloma

## Abstract

**Objective:**

To evaluate the clinical efficacy of continuous veno-venous hemofiltration (CVVH) combined with hemoperfusion for the treatment of multiple myeloma (MM) complicated with acute kidney injury (AKI).

**Methods:**

Medical records of 73 patients with MM complicated with AKI admitted to the First People’s Hospital of Huzhou from January 2019 to January 2021 were retrospectively analyzed. According to the treatment records, 35 patients received simple chemotherapy (control group), and 38 patients received CVVH combined with HP on the basis of chemotherapy (observation group). We compared the clinical efficacies, renal function indexes, and the serum globulin and erythrocyte sedimentation rate (ESR) values between the two groups.

**Results:**

After the treatment, the total efficacy of the observation group was significantly higher (81.58%) than that in the control group (57.14%; *p* <0.05). Serum cystatin C (CysC), urea nitrogen (BUN), β_2_ macroglobulin (β_2_-MG) and creatinine (SCr) levels were significantly lower in the observation group than in the control group (*p* <0.05). Serum globulin level and ESR values in the observation group after the treatment were also significantly lower than in the control group (*p* <0.05).

**Conclusions:**

The outcomes of patients with MM complicated with AKI treated with CVVH and hemoperfusion differ significantly from those of the patients treated only with CVVH. Combining CVVH and hemoperfusion helps to improve the efficacy of the treatment, promotes renal function recovery, and improves the levels of serum globulin and ESR.

## INTRODUCTION

Multiple myeloma (MM) is a very common blood system disorder in which patient’s malignant plasma cells clonally secrete large amounts of immunoglobulins, leading to multi system organ lesions and a variety of complications. Acute kidney injury (AKI) is a common severe complication of MM,^1^ and is mainly manifested by edema, fatigue and nausea. After diagnosis, an anti-tumor treatment should be carried out promptly, by inhibiting the secretions of abnormal monoclonal immunoglobulin (mIG).^2^,^3^

Chemotherapy is the main strategy to treat patients with MM complicated with AKI. However, while it can effectively kill tumor cells, it has insufficient therapeutic effect on the renal function.^4^ With the continuous development of blood purification technologies, a blood purification scheme has been applied to MM/AKI patients with remarkably beneficial results.^5^ Continuous veno-venous hemofiltration (CVVH) combined with hemoperfusion is a common combination for blood purification. CVVH effectively removes the inflammatory mediators and maintains hemodynamics and hemoperfusion occurs through extracorporeal circulation. Resin and activated carbon in the system bind toxic components in the blood and purify it. This combined treatment effectively alleviates the inflammatory reaction, inhibits the progression of the disease, and promotes improvement.^6^

The objective of this study was to evaluate the clinical efficacy of CVVH combined with hemoperfusion for the treatment of patients with MM complicated with AKI. We used clinical records of 73 patients to collect data for our analysis. Our results may provide useful evidence for the physicians to improve treatment outcomes in patients with MM complicated with AKI.

## METHODS

We retrospectively analyzed medical records of 73 patients (48 men and 25 women; mean age of 63.60±8.25 years) with MM complicated with AKI that were admitted to the First People’s Hospital of Huzhou from January 2019 to January 2021. Based on the treatment records, 35 patients that received simple chemotherapy were set as the control group, and 38 patients that received CVVH combined with HP in addition to chemotherapy were set as the observation group.

This study was approved by the First People’s Hospital of Huzhou Ethics Committee (Approval No.: LL20220506; Date: 06 May, 2022)

### Inclusion criteria:

- Patients with diagnosis confirmed by pathology and imaging results meeting the diagnostic criteria for MM complicated with AKI;^7^
- Patients older than 18 and younger than 75 years;
- Patients who had received CVVH combined with hemoperfusion for the first time;
- Patients with complete medical records;

### Exclusion criteria:

- Patients who were pregnant or breastfeeding;
- Severely ill patients requiring a kidney transplant;
- Patients presenting with combined severe organ dysfunction, cancer, or mental or coagulation disorders.

### Chemotherapy:

The patients were administered a 1-1.3 mg/m^2^ bortezomib intravenous injection (Jiangsu Hansoh Pharmaceutical Group, H20173307) on treatment days 1, 4, 8, and 11. Additionally, the patients received 9 mg/m^2^ of melphalan oral tablets (Glaxo Wellcome, H20040125) and 60 mg/m^2^ of prednisone acetate oral tablets (Tianjin Lisheng Pharmaceutical, H12020123) during the first 7 days of each of the four consecutive 21-day treatment cycles.

### CVVH combined with hemoperfusion:

A multi Filtrate 3MUG7641 extracorporeal blood therapy machine, a Fresenius AV 600 polysulfone membrane hemofilter (Membrane area 1.4m^2^, filtration coefficient 40ml/h·mm Hg), and an HA330 disposable resin infuser produced by Zhuhai Jianfan were used. An internal jugular vein or femoral vein of each patient was punctured with a jugular double-lumen catheter. The CVVH treatment was performed after two hours of blood perfusion, the blood flow parameters were set to 200-250 mL/minute, unfractionated heparin was used for anticoagulation, heparin infusion was continued at 300-700 U/hour to maintain activated partial thromboplastin time at 50% greater than normal.

### Basic clinical information of the patients and efficacy evaluation indicators:

1) We assessed the treatment efficacy of the two groups according to the standards established by the International Myeloma Working Group, and treatment outcomes were classified into categories. Complete remissions (CRs): cancerous tissue has completely disappeared and remains without recurrences for more than 30 days; Partial responses (PRs): the highest diameter of the lesion tissue and the product of the highest perpendicular diameters are reduced by ≥50%, while other lesions are not increased for at least 30 days in stable disease (SD), the reduction rate of the product of the two largest perpendicular diameters of the lesion is less than 50%, the increase rate is ≤25%, or the appearance of new lesions is stable; Progressive disease (PD): size increase of lesions larger than 25%, or appearance of new metastases. Total efficacy rate = (CR+PR)/total number of people × 100%.^8^

2) Levels of serum cystatin C (CysC), urea nitrogen (BUN), β_2_-microglobulin (β_2_-MG) and creatinine (SCr) were determined using 3-mL cubital venous blood samples and a Hitachi 7020 automatic biochemical analyzer. 3)Serum globulin level was measured by immunoturbidimetry, and the ESR was measured using the free sedimentation method from 3-milliliter cubital venous blood samples (centrifuged at 3000 rpm × 15 minutes in a 10-centimeter radius centrifuge). All reagents were purchased from Roche Biological Reagent Company and used following the manufacturer’s instructions.

SPSS 22.0 software was used to process the statistical data. Non-rank count data were expressed as numbers and percentages n(%) and were tested by the *χ^2^* test. Measurement data were expressed as means plus standard deviations () and compared using the t test. P<0.05 was considered statistically significant.

## RESULTS

Medical records of 73 patients were included in this study (35 in the control group and 38 in the observation group). We found similar background data between the two groups (*p* >0.05; Table-I). After one year of follow-up, after the treatment, there were 14 complete remissions and 19 partial remissions in the observation group, with a total effective rate of 81.58%. There were nine complete remission and 11 partial remissions in the control group, with a total effective rate of 57.14%. Thus, the efficacy in the observation group was significantly higher than that in the control group (*p* <0.05; Table-II). There was no difference in the serum levels of CysC, BUN, β2-MG and SCr levels between the two groups before the treatment (*p* >0.05). After the treatment, the observation group had significantly serum levels of all biochemical indexes compared to the control group (*p* <0.05), as shown in Table-III. We found no significant differences in serum globulin levels or the ESR rate between the two groups before the treatment (*p* <0.05; Table-IV).

**Table-I:** Comparison of general information between the two groups [*n* (%),*χ̅*±*S*].

Groups	*n*	Gender (Men/Women)	Age (Year)	Disease Classification				

				IgA type	IgD type	IgG type	Non-Secreting Type	Light chain type
Control group	35	21/14	62.20±8.93	7	6	8	5	9
Observation group	38	27/11	64.89±7.45	10	7	11	6	4
*χ^2^*/*t*	-	0.988	1.403	2.976				
*p*	-	0.320	0.165	0.562				

**Table-II:** Comparison of clinical efficacy between the two groups [*n* (%)].

Groups	*n*	Clinical efficacy				Total effective rate

		CR	PR	SD	PD	
Control group	35	9 (25.71)	11 (31.43)	12 (34.29)	3 (8.57)	20 (57.14)
Observation group	38	14 (36.84)	19 (50.00)	4 (10.53)	1 (2.63)	31 (81.58)
*χ* ^2^	-	-	-	-	-	8.111
*p*	-	-	-	-	-	0.044

**Table-III:** Comparison of renal function variables between the two groups (*χ̅*±*S*).

Groups	*n*	CysC (mg/L)		BUN (mmol/L)		β_2_-MG (mg/L)		SCr (μmol/L)	

		Before treatment	After treatment	Before treatment	After treatment	Before treatment	After treatment	Before treatment	After treatment
Control group	35	3.33±1.36	1.66±0.81^a^	12.45±2.83	6.18±1.37^a^	5.58±1.16	2.60±0.87^a^	120.11±19.08	84.57±15.79^a^
Observation group	38	3.47±1.30	0.88±0.41^a^	12.19±2.82	4.65±1.02^a^	5.88±1.45	1.66±0.67^a^	117.60±18.59	74.23±12.07^a^
*t*	-	0.457	5.257	0.031	5.374	0.906	10.599	0.569	3.121
*p*	-	0.649	<0.001	0.975	<0.001	0.368	<0.001	0.571	0.003

***Note:***

a Compared with this group before treatment *p* < 0.05.

**Table-IV:** Comparison of serum globulin and ESR values between the two groups (*χ̅*±*S*).

Groups	*n*	Serum globulin (g/L)		ESR (mm/h)	

		Before treatment	After treatment	Before treatment	After treatment
Control group	35	40.04±9.58	29.87±7.16^a^	41.47±8.17	29.98±8.54^a^
Observation group	38	42.17±9.97	23.26±4.73^a^	40.80±9.00	22.37±6.41^a^
*t*	-	0.004	4.687	0.333	4.275
*p*	-	0.997	<0.001	0.740	<0.001

***Note:***

a Compared with this group before treatment *p* < 0.05.

## DISCUSSION

This study evaluated clinical effect of CVVH combined with HP in the treatment of MM patients with AKI. We showed that this treatment scheme was associated with the improved curative effect, promoted the recovery of renal function, and improved the level of serum globulin and erythrocyte sedimentation rate. Naqvi R et al.^9^ has studied the epidemiological trend of community-acquired acute renal injury in Pakistan for up to 25 years, and showed that older age, coagulation disorders, liver dysfunction, hyperkalemia, need for mechanical ventilation and multiple organ failure were all predictors of high mortality in AKI patients. Our study monitored the recovery of renal function and the changes in serum globulin and erythrocyte sedimentation rate.

Liu P et al.^10^ randomly divided 91 patients with acute paraquat poisoning into hemoperfusion group (49 cases) and HP-CVVH group (42 cases), and showed that the combined therapy of hemoperfusion and CVVH can prevent the patients with acute paraquat poisoning from early death and prolong the survival. Similarly, Gao Y et al.^11^ also showed that the combined treatment of HP and CVVH can prevent early death and prolong survival after acute paraquat poisoning. Premuzic V et al.^12^ compared the effects of plasma exchange and bortezomib for treating patients with MM and AKI, and found that plasma exchange can reduce the amount of free light chains in blood. Studies have shown that prompt treatment of patients with MM and AKI improves their prognosis by reversing kidney disease.^13^

Chemotherapy is important for patients with MM complicated with AKI and it is effective to some extent. However, during the early stages of chemotherapy, free light chain immunoglobulin, produced after the degradation of the mIG in the blood, may cause persistent damage to renal tubular epithelial cells. Therefore, blood purification is an important clinical intervention to treat kidney disease. Plasma exchange removes pathological proteins while retaining healthy immune globulins, and significantly prolongs the survival of patients.^14^,^15^

Combined with chemotherapy, plasma exchange can promote renal recovery and prolong the survival. In our study, patients with MM and HP were treated with CVVH combined with HP in addition to chemotherapy. Our results showed that the total efficacy of the observation group was higher than that of the control group (*p*<0.05), suggesting that the application of CVVH combined with hemoperfusion for the treatment of patients with MM and AKI helps to improve treatment efficacy. Our findings are similar to those of Pendón-Ruiz de Mier MV et al.^16^ that showed that the CVVH treatment regimen provides high solute clearance to maintain stable hemodynamics, promotes tissue oxygen metabolism by removing inflammatory mediators, and provides patients with adequate nutritional support to maintain water-electrolyte balance.

Blood perfusion treatments produce non-selective removal of inflammatory mediators, blocking cytokine cascade reactions, removing toxins from plasma, regulating the immune response, and relieving the degree of tissue and organ damages. Together, this allows to improve the overall curative effect of chemotherapy.^17^

Serum CysC, BUN, β_2_-MG, and SCr are all important indicators of renal function. Bottari G et al.^18^ reported marked renal function improvements in children with sepsis-related AKI treated by CVVH combined with hemoperfusion. In our study, levels of serum CysC, BUN, β_2_-MG and SCr in the observation group after the treatment were lower than those in the control group (*p* <0.05), suggesting that CVVH combined with hemoperfusion promoted the recovery of the renal function. The results of Bottari G et al^18^ are consistent with ours.

When CVVH is used in combination with hemoperfusion, it fully absorbs and removes endogenous and exogenous toxic substances, removes inflammatory factors, and replaces fresh blood into the patient’s body with enough normal immunoglobulin to promote adequate function. Moreover, this treatment regimen increases the effectiveness of chemotherapy, diminishes levels of CysC, BUN, β_2_-MG, and SCr, and relieves the degree of renal impairment.^19^

In our study, the levels of serum globulin and ESR in the observation group after the treatment were lower than those in the control group (*P*<0.05), indicating that CVVH combined with hemoperfusion can also improve serum globulin and ESR levels in MM patients with AKI. Combination of CVVH and hemoperfusion can reduce the expression of inflammatory factors, improve renal and endothelial cell tissue functions, enhance vascular permeability, improve microcirculation and tissue perfusion, promote the stability of hemodynamic indexes, improve oxygenation, and reduce the levels of serum globulin and ESR.^20^

This study hopes to provide some suggestions for clinicians, treating MM/AKI patients. Improved treatment effect as a result of incorporating CVVH and HP in the treatment regimen of MM/AKI patients may potentially reduce the average length of hospitalization, improve patient’s quality of life, and reduce the financial burden on healthcare system by lowering the relative cost of the treatment.^21^

### Limitations:

We only analyzed the patients with MM complicated with AKI in our hospital. Our sample size was small, with only few observation indicators. We provided no long-term follow-ups to evaluate the survival and long-term efficacy of the treatment. Further large sample size multi-center study should be carried out to verify our research results.

## CONCLUSION

CVVH combined with hemoperfusion is effective for the treatment of MM patients with AKI, helping to improve the curative effect, promote the recovery of the renal function, and improve serum globulin and ESR levels.

### Authors’ contributions:

**JH and** **FQ** conceived and designed the study.

**HZ**, **XS and XL** collected the data and performed the analysis.

**JH and FQ** were involved in the writing of the manuscript and are responsible for the integrity of the study. All authors have read and approved the final manuscript.
